# When it pays to cheat: Examining how generalized food deception increases male and female fitness in a terrestrial orchid

**DOI:** 10.1371/journal.pone.0171286

**Published:** 2017-01-31

**Authors:** Ryan P. Walsh, Helen J. Michaels

**Affiliations:** 1 Department of Conservation, The Toledo Zoological Society, Toledo, Ohio, United States of America; 2 Department of Biological Sciences, Bowling Green State University, Bowling Green, Ohio, United States of America; Indian Institute of Science, INDIA

## Abstract

**Background:**

Experimental manipulations of floral nectar in food deceptive species can reveal insights into the evolutionary consequences of the deceptive strategy. When coupled to pollen tracking, the effects of the deceptive pollination syndrome on both male and female reproductive success may be quantified. Attraction of pollinators in deceit-pollinated species often relies on producing a conspicuous floral display which may increase visibility to pollinators, but in-turn may increase within plant selfing.

**Methodology:**

To understand the role of deception in Orchidaceae reproduction we studied *Cypripedium candidum*. All species of the *Cypripedium* genus employ a generalized food deceptive pollination strategy and have been suggested as a model system for the study of pollinator deception. We conducted a nectar addition experiment that randomly assigned the four plants closest to a transect point to receive one of four histochemical dyes. Two individuals selected for nectar addition in each of altogether 25 blocks received 2μl of 25% sucrose solution in the labellum of each flower, while two others received no artificial nectar. Number of fruits produced, fruit mass and fruit abortion were scored at the end of the four-month experiment.

**Results:**

Nectar addition increased (p<0.0001) self-pollination and pollen discounting by nearly 3x, while plants not receiving nectar had greater (p<0.0001) numbers of non-self pollinia deposited and lower rates of pollen discounting. There was a non-significant (p = 0.0645) trend for deceptive plants to set more fruit, while presence of nectar did not affect pollen export.

**Conclusions:**

This study demonstrates the adaptive advantages of food deception by showing a concurrent reduction in particular male and female functions when a food reward is restored to a deceptive flower. We found generalized food deception to not only decrease inbreeding depression in the system, but concurrently have no effect on pollinator attraction and fruit set when compared with rewarding flowers.

## Introduction

Plants have evolved many strategies to maximize pollen transfer efficiency [1], including the evolution of large floral displays. Large floral displays attract more visitors by providing a larger visual presence and a greater concentration of potential food resources [2,3]. Increased floral display size can arise through increases in flower size, increases in the number of open flowers or synchronous flowering. Increasing the floral display size should increase attractiveness to pollinators and visitation rate [1,4–9]. However, while producing multiple flowers or inflorescences on a single plant increases visitation, it may simultaneously increase the probability of intra-individual self-pollination, or geitonogamy, which can reduce fitness in a highly outcrossing species [2,3,10–16].

Deceptive pollination, specifically food deception, is a pollination strategy in which the flower provides floral cues indicating a food reward while not actually providing that reward [17–19]. Non-rewarding flowers are found in 146 genera from 33 flowering plant families [20], but are most prevalent in the Orchidaceae. Of the approximately 7500 food deceptive plant species, approximately 6500 belong to this family [21], where nearly one-third of all species employ this strategy [18]. Sun *et al*. [14] and Kindlmann & Jersáková [3] as well as many others [22–24] have postulated that deceptive orchids with multiple stems avoid the problem of increased geitonogamy because bee visitors tend to visit fewer flowers on the same plant when no reward is provided [25,26]. Floral display size, specifically number of flowers, is positively correlated with pollination success in numerous food deceptive orchid species [27–29]. Deceptive pollination has fascinated biologists since its discovery by Sprengel in 1793 [30] and Darwin’s 1862 treatise on orchid pollination [31]. However, the role deception plays in the reproduction of deceptive plants has remained enigmatic [32] despite the exhaustive 200 years of research on food deceptive species. Although this pollination strategy can reduce geitonogamy in multi-flowered individuals by causing pollinators to flee non-rewarding patches [3,14,22–24,32,33], it often results in reduced floral visitation and pollination when compared to non-deceptive relatives [25,26], and relies on newly emergent or naïve insects for effective pollination services [33]. Furthermore, as discussed by Johnson *et al*. [34], the presence of large numbers of rewardless species presents a conundrum in understanding the evolution of orchid pollination as numerous studies have shown increased pollination and fruiting success as well as visitation time by pollinators [35] in rewarding orchids.

Various other hypotheses have been proposed to explain the costs and benefits of the deceptive pollination strategy [32]. Aside from larger floral displays, many deceit-pollinated plants may also benefit from the close proximity of other nectar providing plants [33,34]. In this case, a flower may be discovered via the instinctive foraging behavior of a nectar-seeking insect while avoiding the physiological cost of producing nectar and the potential reproductive cost of geitonogamous pollination. It has also been suggested that deceptive orchid pollination is a type of Batesian mimicry between the deceptive orchid and neighboring nectar producing plants [36]. Schiestl [32] further posits that deception may encourage pollen flow over longer distances.

Understanding the role of deception in orchid evolution requires a detailed examination of both male and female function. Deception that leads to geitonogamy serves to reduce female function, but also male function by reducing the quantity of pollen available for export to other plants, also known as pollen discounting [24]. While the pollinium structure of orchids makes pollen removal easy to score [37], removal alone has shown variable success in estimating pollen export [38–42]. Peakall [4] was first to examine the fate of orchid pollinia using histochemically stained pollinia. At the time of Kropf & Renner’s 2008 review [21], the fate of pollen had been directly surveyed in relatively few orchid species [21]. Maximum pollinia transport distances have varied widely from 6.9m in an *Andrena* pollinated species [21] to 76m in a hawkmoth-pollinated species [43]. Even fewer studies have directly addressed the role of deception through nectar addition studies [23,24,44–46].

Nectar addition studies seek to understand the reproductive benefits of deceptive pollination by supplementing nectar and quantifying the resultant change in pollination and fecundity. The addition of nectar to non-rewarding species in these studies resulted in increased pollinator foraging times, increased pollinia removal and increased rates of self-pollen deposition [44]. Working with the food deceptive orchid *Anacamptis morio*, [45] found that nectar supplementation increased both average foraging time on an individual plant and self-pollination. Previous studies have focused primarily on orchids pollinated by *Bombus* spp. [23,24,45,46] as well as moths [45] and flies [44]. The size, average flight distance and general foraging behavior of a pollinator would be expected to alter the corresponding response to deception with larger, more intelligent or more social animals less likely to succumb to the deceptive mechanism numerous times, thereby drastically decreasing the probability of successful pollination [47–51]. Furthermore, density of conspecific potential mates may be critical in the success of the deceptive syndrome. Low densities of conspecifics within the average flight range of the flower visitor, coupled with animals that learn to avoid the floral deception would result in a further reduction in probability of pollination in the already highly pollination limited system [27].

This paper examines the effects of deception on male and female function of multiple-stemmed flowering perennials, using the slipper orchid, *Cypripedium candidum* (subfamily Cypripidiodieae). The *Cypripedium* genus has been proposed as a model system for the study of orchid reproduction, conservation and the evolution of deception due to its unique trap-like floral design, the presence of deception throughout the entire genus, consistent pollinator limitation, low amount of pollinator fidelity and the abundance of self-incompatible species [52]. The trap-like structure in the flower ensures an animal passing through the flower will receive one of two pollinium, thereby allowing quantification of visitation rates with a high degree of accuracy. Although several studies have examined the role of nectar addition on deceptive orchid species, none have specifically focused on the *Cypripedium* genus and these unique attributes. Although the genus *Cypripedium* has evolved a non-rewarding flower that should reduce within-flower selfing, the pollinator limitation observed in the genus [29,53–55], is likely to drive selection towards individuals that produce multiple flowering stems with synchronous flowering within an individual. This may increase attractiveness to pollinators at the cost of increasing the probability of between-flower selfing (geitonogamy). We hypothesize pollination by deceit increases fitness by decreasing intra-individual pollen transfer and increasing pollen export rates. This study specifically addresses the following questions: 1. Does the presence of a nectar reward affect pollinia deposition? 2. Does nectar presence influence pollinia transport distances? 3. Does the presence of nectar increase geitonogamous pollination? and 4. Do surrounding conspecific and allospecific nectar-producing species influence both male and female function? We expected the presence of a nectar reward would increase foraging time on a plant, and therefore predicted an increase in the probability of geitonogomous pollination [3]. Furthermore, on plants without added nectar, pollen removal and deposition should be dependent on attractiveness to the pollinator and therefore display size. The presence and proximity of nearby nectar producing plants should draw pollinators into the vicinity and thereby increase fruit set in the system [56].

## Materials and methods

*Cypripedium* species are deciduous, terrestrial orchids that develop from a subterranean rhizome [57]. *Cypripedium candidum* Muhl ex Willdenow, the Small White Lady’s Slipper, with yellow-green lateral sepals and petals with a white labellum spotted with purple [57], occupy calcareous prairies as well as fens and limestone barrens [58]. *C*. *candidum* is highly dependent on open areas with full sun and, as with many prairie species, populations begin to decline with the invasion of woody plants [59]. The plants occur as single plants or large clumps (1–12 vegetative stems or ramets) typically bearing a single flower per inflorescence that accommodates a single pollinium when fertilized. Each flower has two anthers, each containing a single pollinium. A pollinator entering the flower first encounters the stigma followed by one of two anthers during which the pollinator picks up a single pollinium. Upon visiting a second flower, the pollinator may deposit pollen grains, or an entire pollinium on the stigmatic surface. Multi-parental pollinations, or the presence of pollinium from multiple sires on a single pollinator have been recorded in the larger *Bombus spp*. pollinated C. *parviflorum* var. *pubescens* [60], but have not been recorded or noted in the smaller *C*. *candidum* where both the flower and pollinators are approximately ¼ the size of the other system. The floral architecture, coupled with the short flowering time (~2 weeks) and food deceptive strategy, severely constrains opportunities for pollination. Although *C*. *candidum* do not produce a nectar reward (like all taxa in the genus *Cypripedium*), they are visited by predominately small (4–6 mm long) Andrenidae and Halictidae bees [61–63]. The large size of one pollinium relative to these small bees makes it difficult for a pollinator to carry more than one pollinium at a given time. During field observations of pollinators we found the pollinium to be removed from the stamen in its entirety and the entirety of the pollinium deposited on the stigma of the next visited flower. During a preliminary study we estimated pollinator density as >0.5 animals per m^2^ per 5 minute sampling period and more than 80% of recorded pollinations contained entire pollinium.

To understand the role of deception in *C*. *candidum*, we conducted a nectar addition experiment from May-August 2011 at Resthaven Wildlife Area in Castalia, Ohio (GPS coordinates available upon request) under the permission of the Ohio Division of Natural Resources (land owner). Resthaven is one of three locations in Ohio where *C*. *candidum* occurs, and has an estimated 6000 plants in an actively managed prairie area (approximately 900 hectares) with wooded areas intertwined (pers. comm. J. Windus, Ohio Department of Natural Resources). The prairie is maintained through early spring controlled burns approximately every three years. Resthaven has 16 populations of *C*. *candidum*, of which we were permitted to work in four. This study was carried out in the largest of those populations containing roughly 450 individuals (pers. comm. J. Windus, Ohio Department of Natural Resources). Six 80m transects were distributed equally across this population, with the initial transect location randomly chosen. At 20m intervals along each transect, we selected four similar-sized plants closest to the transect within a 1 m^2^ quadrat by finding the plant closest to the center of the quadrat, and then locating three other plants within the quadrat with the same number of flowers and stems to control for differences in size and the potentially confounding factor of resource reallocation [64,65]. Individual stems (ramets) within 20cm of each other were considered as part of a single individual (genet) based on previous literature in the genus and species [66–68]. We chose the 20m interval between quadrats to ensure we could differentiate between pollina sources in quadrats when searching for stained pollinia deposited on non-experimental plants based on previously reported pollination distances of 6-8m in similar pollinator assemblages of andrenids and halictids [21]. A total of 100 plants were examined during this experiment, consisting of 4 plants within each quadrat across a total of 25 quadrats with 50 plants per nectar treatment.

Plants were selected for experimental manipulation before anthesis, and buds were covered with a pollinator exclusion bag to prevent pollinia removal prior to the beginning of the experiment. At each group of four plants, a randomized block design was employed to randomly assign which of four histochemical dyes (Neutral Red, Gentian Violet, Fast Green and Methylene Blue, all at 1% solution) were applied to the pollinia of each plant, with two plants selected to receive nectar. Fifty individuals selected for nectar addition received 2μl of 25% sucrose solution in the labellum of the flower. Nectar was removed and replaced every two days. Following the framework established by Johnson *et al*. [24], 1–2 μl of histochemical dye of one of the four different colors were injected onto the pollinia of study orchids (total N = 100) to color code the pollinia using a P10 micropipette (P10 Pipetman^®^, Gilson Inc., Middleton, WI, USA) approximately 24 hours after flower opening. Stain was allowed to sit on each pollinium for two minutes prior to gently blotting the excess stain with a clean paper towel. The procedure was repeated in the case of inadequate staining. We checked stain for color maintenance during data acquisition, but re-staining was not required during the short flowering duration (< 2 weeks). The other 50 individuals received no additional nectar, but had stain injected onto their pollinia using the same procedure.

We scored plants for pollen receipt or removal from the stigmatic surface every 2 days throughout the flowering period (15 days). The presence of pollinia containing either non-dyed pollinia or dye of a different color on a stigma was scored as an outcrossing event, while presence of deposited pollinia dyed with the same color initially applied to a plant’s pollinia was scored as a selfing event. Additionally, we assessed the proximity of surrounding nectar-producing co-flowering plants by measuring and identifying the distance to the three nearest nectiferous plants. The plants in flower at the time of the study and included in this analysis were: *Maianthemum racemosum*, *Fragaria vesca*, *Viola* spp., and *Hypoxis hirsuta*. If a pollinium had been removed, all plants within a 10m radius were searched for the dyed pollinium. The distance to the nearest likely (dyed with the appropriate color) pollen source plant was measured and recorded for all deposited pollinia. Capsule development was recorded one month after floral dehiscence (June) as well as at maturity in August when all fruits were collected and scored for insect damage. Fruit abortion was scored as the number of fruits initiated in May minus the number matured in August. Fruit abortion rate was then calculated by dividing the number of fruits aborted by the total number of fruits produced. Mature capsules were dried at 60°C for three days prior to obtaining the masses for the capsule and seeds. Seed mass was measured to the nearest hundred-thousandth of a gram on a Mettler AE-240 scale (Mettler-Toledo Inc., Columbus, OH, USA). We estimated the effect of inbreeding depression (δ) on female reproductive success by calculating the mean seed production of mature capsules on both self and outcross pollinated plants as δ = 1- (ω_s_/ ω_x_) where ω_s_ is the seed mass produced by selfing and ω_x_ is the seed mass produced by an outcross event [69]. Pollen discounting (δ_p_), the reduction in male outcross success that results from self-fertilization, was estimated for each plant as:

$$\delta \text{p}=\frac{S}{R}$$

where S = the number of self pollinia deposited on the plant and R = the number of pollinia removed [68]. Pollen discounting rates were compared between the two treatments using a Mann-Whitney U test.

Analyses were performed using JMP for Mac v.9.0.2 (SAS Institute, California, USA). The components of female reproductive success (FRS, measured as receipt of self, out-cross pollinia and total pollinia received, percent fruit set {number of fruits divided by number of flowers, F_*p*_}, and fruit abortion) were assessed for meeting assumptions of ANOVA, after which the percent data was transformed using arc-sine square root. We analyzed each dependent variable using a Standard Least Squares ANOVA with quadrat, number of stems, number of flowers, treatment and the average distance to the three nearest nectar-producing plants as predictors. The effects of pollinium type (selfed or outcross), number of flowers and number of stems on the seed mass of plants that set fruit was checked for normality and homogeneity of variance and analyzed using a Standard Least Squares ANOVA. The difference in seed mass between selfed and outcrossed capsules was compared with a paired t test. Total pollinia received never exceeded 1 on an individual flower. Male reproductive success (MRS) was calculated as a variation of (*F*_*r*_*/F*_*tot*_) x 100 where *F*_*r*_ is the number of flowers found with one or both pollinia removed and *F*_*tot*_ is the total number of flowers in the study. To estimate pollination efficiency (PE), female reproduction was quantified as percent fruit set (F_*p*_) and PE was then calculated as F_*p*_/ *F*_*r*_. As discussed in Scopece *et al*. [27], this calculation results in a range of values between 0 and 1, with 1 representing high efficiency and 0 the lowest efficiency. Comparisons between nectar and non-nectar treatments were made for MRS and PE using a Wilcoxon rank sum test.

## Results

Plants included in the study had an average of 3.01 (SE = 0.13, range 2–8) stems and 2.42 (SE = 0.06, range 2–4) flowers per plant. The population density of *C*. *candidum* at Resthaven Wildlife Area (WA) was previously estimated at 3.26 plants per meter^2^ (range = 1–9 plants/m^2^; SD = 2.68) [29]. Of the total of 484 pollinia that were stained, 103 (21.3%) were removed during this experiment. Male reproductive success did not differ between treatments, with 52 pollinia removed from non-nectar treated plants and 51 pollinia removed from nectar treated plants (MRS = 44.0 for no nectar, MRS = 44.33 for nectar, W = 1267.5, p = 0.90). However, pollen discounting significantly differed between treatments (W = 758.5, p < 0.001), where 52% of pollinia removed in the nectar treatment resulted in geitonogamy (δ_p_ = 0.52), while only 17% of pollinia removed resulted in selfing in the no-nectar treatment (δ_p_ = 0.17).

**Fig 1:**
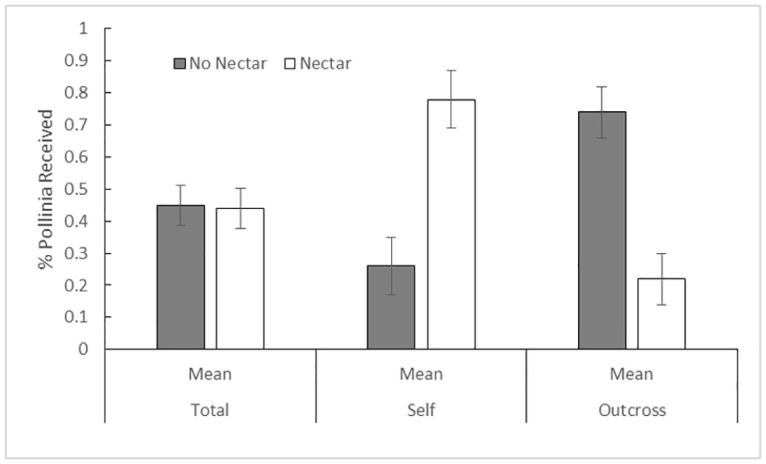
Comparison of pollinia receipt by treatment using standard least squares ANOVA. There was no difference in total receipt of pollinia between treatments (F_1,99_ = 0.04, p = 0.83, ANOVA F_28,99_ = 1.18, p = 0.27, R^2^ = 0.31); however, plants receiving nectar had significantly more self-pollination events (F_1,99_ = 15.76, p < 0.001, ANOVA F_28,99_ = 1.82, p = 0.021, R^2^ = .041) and fewer outcross events (F_1,99_ = 18.12, p < 0.0001, ANOVA F_28,99_ = 1.61, p = 0.05, R^2^ = 0.38) than flowers that did not receive supplemental nectar. Error bars represent standard errors.

Nectar did not influence pollinia receipt (F_1,99_ = 0.06, p = 0.81, R^2^ = 0.31; S1 Table, Fig 1). However, we found a significant effect of nectar addition on self pollinia receipt, (F_1,99_ = 12.57, p = 0.0007, R^2^ = 0.41; Fig 1). The number of flowers on a plant had a significant effect on total pollinia receipt (F_1,99_ = 4.22, p = 0.043) and self pollinia receipt (F_1,99_ = 4.17, p = 0.044), but not % outcrossed pollinia received (F_1,99_ = 0.91, p = 0.34)(S1 Table). Quadrat, number of stems and the average distance to the three nearest conspecific neighbors had no effect on pollinia receipt. In this relatively dense population, the mean distance of a nectar producing plant from a flowering *C*. *candidum* was 21.8 cm (SE = 1.23).

Nectar addition had a strong effect on mating system. Of the plants receiving nectar, 78% of pollinia deposited on the stigmatic surface of a plant’s flowers were from selfing, while plants that did not receive nectar received selfed pollinia only 26% of the time. Additionally, the presence of nectar significantly reduced the probability of a plant receiving outcrossed pollen (F_1,99_ = 15.6, p = 0.0002, R^2^ = 0.38). Of the plants receiving nectar, 22% of the pollinia were outcrossed, while the plants not receiving nectar had outcrossed pollen deposited on their stigma 74% of the time. An increase in display size was also significantly associated with increased number of pollinia received (F_1,99_ = 4.22; p = 0.043, R^2^ = 0.31) and receipt of self pollinia (F_1,99_ = 4.17; p = 0.044, R^2^ = 0.41), but not receipt of outcross pollinia (F_1,99_ = 0.91; p = 0.34, R^2^ = 0.38). Color of the dye, transect and quadrat location of the study plants did not significantly affect fruit set or the receipt of pollinia. The mean pollination efficiency of non-nectar plants was higher at 0.55 compared to that of plants receiving nectar (PE = 0.39); however, there was no significant difference between the treatments (W = 1477.5, p = 0.085).

**Fig 2:**
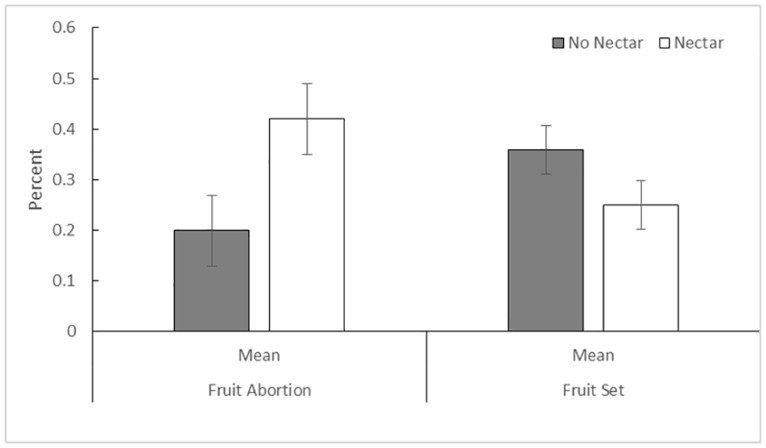
Comparison of the effect of treatment on average female reproductive success of study plants using standard least squares ANOVA, blocked by quadrat. Plants that received nectar had an increased rate of fruit abortion (F_1,99_ = 4.58, p = 0.035) (ANOVA F_3,51_ = 1.48, p = 0.23, R^2^ = 0.42), but no difference in fruit production (F_1,99_ = 2.82, p = 0.09, ANOVA F_28,99_ = 1.31, p = 0.17, R^2^ = 0.34). Error bars represent standard errors.

**Fig 3:**
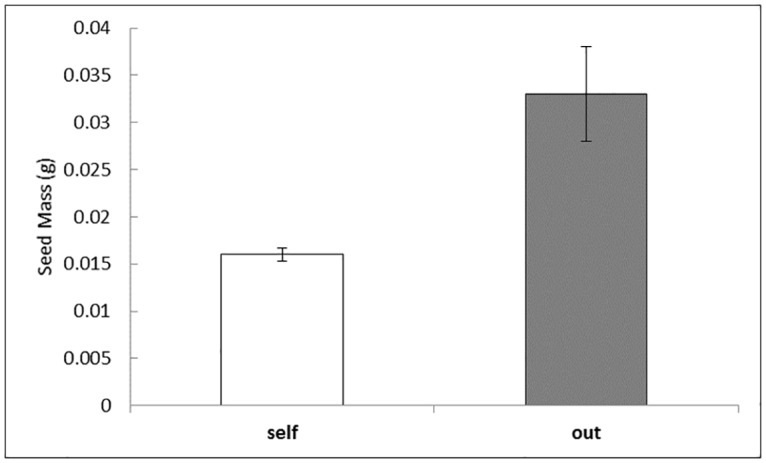
Comparison of seed mass between selfed and outcrossed fruits using a paired t test. Plants that received outcross pollinia had significantly greater seed mass than those receiving selfed pollinia, (paired t(46) = 2.87, SD = 0.032, p = 0.006; mean self = 0.016g, SE = 0.0007, mean outcross = 0.033 g, SE = 0.005). Error bars represent standard errors.

Fruit production (ANOVA F_28,99_ = 1.31, p = 0.17, R^2^ = 0.34, S2 Table) was not significantly affected by nectar addition (F_1,99_ = 2.89; p = 0.09), number of stems (F_1,99_ = 0.05; p = 0.81), or number of flowers (F_1,99_ = 3.27; p = 0.07). A power analysis revealed a required sample size of n = 100 in order to reach the p = 0.05 threshold. Overall, of the 72 flowering stems that matured fruit during the experiment, 46 were from outcross pollinations, while 26 were geitonogamous pollinations. Fruit abortion (ANOVA F_3,51_ = 1.48, p = 0.23, R^2^ = 0.42, S2 Table) was higher in nectar treated plants (F_1,51_ = 4.04, p = 0.049; Fig 2), but was not affected by the number of stems (F_1,51_ = 0.52, p = 0.47), or the number of flowers (F_1,51_ = 0.0, p = 0.99). Seed mass (ANOVA F_3,71_ = 1.63, p = .018, R^2^ = 0.067, Fig 3, S3 Table) of pollinated flowers was not affected by the number of stems (F_1,71_ = 0.17, p = 0.67) or number of flowers (F_1,71_ < 0.001, p = 0.99), but was affected by the source of the pollinia (F_1,71_ = 4.09, p = 0.47). We calculated the effect of inbreeding depression on seed production using the pooled seed mass of known selfed and outcrossed fruits. Most individuals showed increased seed mass when pollinated with outcross pollen (mean δ = 0.512, SD = 0.028), similar to inbreeding depression levels previously found in this system (mean δ = 0.463, SD = 0.31; [29]).

Estimating the distance travelled by pollinia outside of selfing events proved difficult, as only eight of the 103 removed pollini were recovered. Interestingly, all eight originated from non-nectar treated plants, and all were outcross movements to the stigmatic surface of another plant. Of the recovered pollinia, the average distance travelled was quite limited at 40.06 cm (SD = 5.8), while the furthest distance was 75.6 cm. However, the proximity of surrounding nectar-producing plants had no effect on the total number of pollinia received (F_1,99_ = 0.002, p = 0.95), or numbers of self (F_1,99_ = 0.03, p = 0.85) or outcross pollinia received (F_1,99_ = 0.07, p = 0.78, S1 Table), and showed no significant effect on fruit production (F_1,99_ = 0.0002, p = 0.98, S2 Table).

## Discussion

Successful reproduction in any animal pollinated system involves numerous components that must all be met for successful transfer of genetic information. With the strong selection on attracting a suitable vector, it then seems rather perplexing that a pollination strategy would arise in so many lineages that is wholly inefficient when compared to traditional nectar providing species [27,33,70,71]. Several authors have posited that deceptive pollination evolved primarily to increase outcrossing while decreasing geitonogomous pollination [32,33]. In support of this, we observed a natural rate of self-pollination of 26% compared to 78% in the supplemental nectar treatment. Perhaps most surprisingly, we did not see any effects of nectar addition on pollen export, pollination efficiency or fruit set, although we did find a non-significant trend towards increased fruit set in plants that received nectar. Given the preponderance of research suggesting the increased efficiency of rewarding flowers, the lack of increased efficiency seen on nectar supplemented flowers leads us to believe the floral architecture of *Cypripedium* itself may be a constraint on reproduction. While the presence of nectar did increase geitonogomous pollination, thereby reducing male and female reproductive success, it did not result in an overall higher rate of reproduction. These results suggest that nectar reward, like flower number, plays little role in pollinator attraction and that other factors are more limiting to reproduction. If nectar reward were important in the attraction of pollinators we would have expected to see both an increase in geitonogamy as well as an overall increase in fruit set. Instead, it appears that insects attracted to the flower will visit other conspecific flowers, and the presence of nectar only encourages increased intra-plant foraging.

This result is contrary to most deception literature that shows increases in pollination when nectar is present [24,71]. However, the high density of this study population where different, non-rewarding individuals are within cm of the nectar-supplemented plant, may obscure any increased pollination due to nectar reward by chance alone. In a low density population where the next conspecific is beyond the normal foraging range of this small pollinator, we would expect the pollinator to continually return to the known, concentrated food source, thereby increasing geitonogamy. Furthermore, concurrent research [29] showed a strong effect of flower height on pollination rates, which may have had an over-riding influence and may explain why nectar addition had no overall effect on increasing pollination rates. Other research in the closely related *Cypripedium acaule* [72] saw similar effects of floral height. Although this study found no significant effect of number of flowers and number of stems on proportion of fruits produced, we did find increased total pollinia receipt as well as self pollinia receipt with increased flower number. Furthermore, in such a relatively dense population of orchids (3.26 plants/m^2^), it is possible many of the pollinators rapidly learn to avoid *C*. *candidum*, reducing any effects of nectar addition [26,73–75].

Pollen discounting, or the reduction in pollen available for outcross as a result of selfing events [76] may play an important role in explaining the advantage of deceptive pollination. In our study, we observed considerable pollen discounting within the nectar addition treatment. In the nectar restored treatment, 52% of the pollinia found resulted in geitonogamy. Not only does this dramatically increase the chances of inbreeding depression, but it reduces male fitness by decreasing the availability of pollinia that can outcross. Similar results were seen in the deceptive orchid *Anacamptis morio* (Syn. *Orchis morio*) where Johnson, Peter & Ågren [24] estimated that nectar production would result in 39.5% of the removed pollen being deposited on a self-stigma but only 8.6% if nectar were not provided. In the case of orchids, specifically *Cypripedium candidum*, each flower only produces two pollinia, potentially exacerbating pollen discounting further.

Self-pollination often leads to decreased fitness through reduced seed set or seed quality or even fruit abortion [71,77]. We saw no impact of nectar addition on fruit production, but did see an increase in fruit abortion in selfed plants supplemented with nectar, indicating potential inbreeding depression. Other studies have shown similar trends in fruit set with nectar addition in deceptive plants. Johnson & Nilsson [45] noted no increase in fruit set when nectar was added to *Orchis morio* and *Platanthera chlorantha*, as did Ackerman [78] in *Calypso bulbosa*, Smithson and Gigord [46] in *Barlia robertiana* and Smithson [23] in *Anacampstis morio*. This trend has also been noted outside the Orchidaceae [5,79]. We also saw a strong reduction in seed mass following selfing, supporting data previously obtained in the same system through an experiment examining the effects of hand selfing, hand outcross and open pollination on fruit set and seed mass [29]. We observed significant ovule discounting, or reduction in number of ovules available for outcrossing due to fertilization from self pollen [80,81]. In a previous study [29], plants receiving self pollen had an average of 50% lower seed mass than those receiving outcross pollen indicating increased seed abortion during selfing events. Similar results in seed mass reduction and increased fruit abortion were noted in the deceptive orchid *Disa pulchra* when nectar was added [44]. A 50% reduction in seed mass and increase in fruit abortion, multiplied over the long life of a perennial plant such as *C*. *candidum*, would potentially translate to a significant reduction in life-time fitness of the plant.

Although we recovered a very small number of pollinia, most were found at distances less than 1m, suggesting the small body size of the pollinators limits the potential pollinia dispersal distance. Previous studies have found pollinia transport distances to vary widely with pollinator species [21,43] and most have focused on larger pollinators. Kropf and Renner [21] found a maximum transport distance of 6.9m for relatively large *Andrena* bees averaging 8–17 mm long. In contrast, pollinators caught during preliminary trials in our field site averaged 4–6 mm long. Pollen transport distances for the closely related, although larger, *C*. *parviflorum* (syn. *calceolus*) var. *pubescens* were recorded to be 5.2 m by Tremblay [60]. While our study recorded pollinia transport distances over a single season at a single location, maximum foraging distance of bee species has been shown to be directly related to the body size of the pollinator [82]. The small flower entrance and exit of *C*. *candidum* therefore limits the maximum transport distance of its pollinia by limiting the size of pollinators that may enter and leave the flower.

Pollination by deceit has been an area of intense interest to evolutionary biologists, and plants in the Orchidaceae have often been a focus in this research [18], with nearly 1/3 of all orchids employing some type of deceptive strategy [83]. We saw virtually no increase in pollination with the addition of nectar. Furthermore, we observed nearly a 50% reduction in seed mass when pollination of flowers caused selfing, and more than twice the number of fruit abortions with the nectar addition treatment. The presence of nectar in *Cypripedium candidum* led to strong ovule discounting without increasing fruit production. Based on this study and our previous work [29], we can conclude that although the deceptive pollination strategy may entail a significant amount of pollen limitation, the resulting increase in male and female fitness far outweighs the potential reduction in fruit set over the long life of a deceptive plant. Furthermore, removed pollinia are nearly three times as likely to result in selfing when compared to the deceptive system. This does not account for reductions in seed viability and offspring fitness that may come with increased self-pollination and inbreeding depression [84–86]. Orchids that are subject to selfing events have been shown to exhibit inbreeding depression [84,85], especially in deceptive orchids that have evolved with very low levels of selfing [44,86].

## Conclusions

It seems at first counterintuitive that nectar deception, which leads in most cases to lower fruit production, would be maintained so consistently within the Orchidaceae family [87]. Our study demonstrates a strong cost to both male and female fitness that has no doubt been important to the maintenance of food deception in *Cypripedium*. Johnson, Peter & Ågren [24] noted that it was difficult to understand how deceit was maintained in plants with low pollen receipt (<10%). However our data suggests nectar addition does not affect total pollinia receipt or fruit set, thereby making the increase in offspring quality the primary factor in the maintenance of deceit pollination. All studies on deceit in Orchidaceae have examined the effect over a narrow period of time, not completely considering the effects of this strategy over the considerable lifetime of most orchid species. Further studies over longer periods of time that incorporate what is known about deceit pollination, along with other variables affecting reproduction such as seed predation and dormancy, are needed in order to account for the costs and benefits of deception over the life of individuals.

## Supporting information

S1 Table The effect of nectar addition, quadrat, number of stems, number of flowers and the distance of the three nearest non-orchid, nectar-producing neighbors on the percentage of total pollinia (R^2^ = 0.31), percent self pollinia (R^2^ = 0.41), and percent outcross pollinia received (R^2^ = 0.38). Percentage pollinia received calculated as the number of pollinia received divided by the number of flowers on a given plant.
(PDF)

S2 Table The effect of nectar addition, quadrat, number of stems, number of flowers and distance of the three nearest non-orchid, nectar-producing neighbors on percent fruit set (R^2^ = 0.34), and percent fruit abortion (R^2^ = 0.42). (PDF)

S3 Table The effect of pollinia source, number of stems and number of flowers on the seed mass of a capsule, R^2^ = 0.067. (PDF)
