# Supplementary material for: When it pays to cheat: Examining how generalized food deception increases male and female fitness in a terrestrial orchid
Source: PLoS One. 2017 Jan 31;12(1):e0171286. doi: 10.1371/journal.pone.0171286 (PMC5283728; doi:10.1371/journal.pone.0171286)
Supplement: S1 Table — Percentage pollinia received calculated as the number of pollinia received divided by the number of flowers on a given plant. (PDF) [file pone.0171286.s001.pdf]

	Source	DF	Sum of Squares	Mean Square	F Ratio	Prob>F
**% Total Pollinia Received**	Nectar	1	0.01		0.06	0.81
	Quadrat	24	5.31		1.16	0.3
	# of Stems	1	0.003		0.01	0.89
	# of Flowers	1	0.8		4.22	0.043*
	3 Nearest Neighbors	1	0.0005		0.002	0.95
	Model	28	6.3	0.22	1.18	0.27
	Error	71	13.48	0.189		
	Total	99	19.79			
**% Self Pollinia Received**	Nectar	1	1.13		12.57	0.0007*
	Quadrat	24	3.14		1.45	0.11
	# of Stems	1	0.001		0.01	0.9
	# of Flowers	1	0.37		4.17	0.044*
	3 Nearest Neighbors	1	0.003		0.03	0.85
	Model	28	4.61	0.16	1.82	0.021*
	Error	71	6.39	0.09		
	Total	99	11.01			
**% Outcross Pollinia Received**	Nectar	1	1.35		15.6	0.0002*
	Quadrat	24	2.29		1.1	0.36
	# of Stems	1	0.0004		0.005	0.94
	# of Flowers	1	0.079		0.91	0.34
	3 Nearest Neighbors	1	0.006		0.07	0.78
	Model	28	3.91	0.13	1.61	0.05*
	Error	71	6.15	0.08		
	Total	99	10.07			
