# Supplementary material for: When it pays to cheat: Examining how generalized food deception increases male and female fitness in a terrestrial orchid
Source: PLoS One. 2017 Jan 31;12(1):e0171286. doi: 10.1371/journal.pone.0171286 (PMC5283728; doi:10.1371/journal.pone.0171286)
Supplement: S2 Table — (PDF) [file pone.0171286.s002.pdf]

	Source	DF	Sum of Squares	Mean Square	F Ratio	Prob> F
**% Fruit Set**	Nectar	1	0.33		2.89	0.09
	Quadrat	24	3.61		1.29	0.19
	# of Stems	1	0.006		0.05	0.81
	# of Flowers	1	0.37		3.27	0.07
	3 Nearest Neighbors	1	0.00002		0.0002	0.98
	Model	28	4.26	0.15	1.31	0.17
	Error	71	8.23	0.11		
	Total	99	12.5			
**% Fruit Abortion**	Nectar	1	0.77		4.04	0.049*
	# of Stems	1	0.10		0.52	0.47
	# of Flowers	1	0		0	0.99
	Model	3	0.85	0.28	1.48	0.23
	Error	48	9.22	0.19		
	Total	51	10.07			
