# Supplementary material for: When it pays to cheat: Examining how generalized food deception increases male and female fitness in a terrestrial orchid
Source: PLoS One. 2017 Jan 31;12(1):e0171286. doi: 10.1371/journal.pone.0171286 (PMC5283728; doi:10.1371/journal.pone.0171286)
Supplement: S3 Table — (PDF) [file pone.0171286.s003.pdf]

Source	DF	Sum of Squares	Mean Square	F Ratio	Prob>F
**Pollinia Source**	1	0.004		4.09	0.47*
**# of Stems**	1	0.0001		0.17	0.67
**# of Flowers**	1	8.82E-09		0	0.99
**Model**	3	0.004	0.001	1.63	0.18
**Error**	68	0.068	0.001		
**Total**	71	0.07			
